# Complete sequences of pIJ101-based Streptomyces-Escherichia coli shuttle vectors

**DOI:** 10.1099/acmi.0.000893.v3

**Published:** 2024-10-23

**Authors:** Katelyn V. Brown, S. Eric Nybo

**Affiliations:** 1 Department of Pharmaceutical Sciences, College of Pharmacy, Ferris State University, Big Rapids, MI 49307, USA

**Keywords:** next-generation sequencing, pIJ101, plasmid, *Streptomyces*, synthetic biology

## Abstract

High-copy-number plasmids are indispensable tools for gene overexpression studies in prokaryotes to engineer pathways or probe phenotypes of interest. The development of genetic tools for the industrially relevant Actinobacteria is of special interest, given their utility in producing keratolytic enzymes and biologically active natural products. Within the Actinobacteria, *Streptomyces–Escherichia coli* shuttle vectors based on the SCP2* and pIJ101 incompatibility groups are widely employed for molecular cloning and gene expression studies. Here, the sequences of two commonly used pIJ101-based *Streptomyces–E. coli* shuttle vectors, pEM4 and pUWL201, were determined using next-generation sequencing. These plasmids drive the expression of heterologous genes using the constitutive ermE*p promoter. pEM4 was found to be 8.3 kbp long, containing a β-lactamase gene, thiostrepton resistance marker, the *lacZɑ* fragment, a ColE1 origin of replication and the *Streptomyces* pIJ101 origin of replication. pUWL201 was found to be 6.78 kbp long, containing a β-lactamase gene, thiostrepton resistance marker, the *lacZɑ* fragment, a ColE1 origin of replication and the *Streptomyces* pIJ101 origin of replication. Interestingly, the sequences for both pEM4 and pUWL201 exceed their previously reported size by 1.1 and 0.4 kbp, respectively. This report updates the literature with the corrected sequences for these shuttle vectors, ensuring their compatibility with modern synthetic biology cloning methodologies.

## Data Summary

The sequences for pEM4 (GenBank Accession No. MN970094) and pUWL201 (GenBank Accession No. MN992950) have been deposited in the National Center for Biotechnology Information database. Supplementary data have been deposited in Figshare DOI: https://doi.org/10.6084/m9.figshare.27170772.v1.

## Introduction

Next-generation sequencing (NGS) techniques, such as RNA-seq and Illumina whole-genome sequencing (WGS), have transformed the biological sciences via deep sequencing of whole-cell transcriptomic profiles and genomes at significantly reduced cost [1,2]. WGS can also be applied to determine unknown plasmid sequences with great accuracy [3]. As such, WGS is a powerful tool for determining plasmid vector sequences for plasmids that have been in widespread use for several decades but for which no sequence information is available in the literature.

pIJ101 is a high-copy plasmid vector replicated in *Streptomyces lividans* and *Streptomyces coelicolor* at a copy number of ~70–100 copies per cell [4]. Since the first report of its use in the cloning of *Streptomyces* genes in the 1980s, many different derivatives of pIJ101 have been developed, including pWHM3 and pUWL201 [5,6]. These vectors are commonly used to overexpress genes of interest in a wide variety of *Streptomyces* spp. [7,8].

In early reports, cloning via restriction digest and ligation into a multiple cloning site (MCS) was the gold-standard technique for studying gene expression in *Streptomyces*. Yet, due to limitations in the cost and scope of Sanger sequencing, the sequences for pIJ101-based plasmids were unreported during the early decades of actinomycete genetic engineering. However, modern cloning techniques are sequence-specific, including Golden-gate assembly [9], Gibson assembly [10] and even yeast recombination assembly [11], methods for which these plasmid vectors would be incompatible without exact sequencing information. Therefore, in this report, we determined the sequence for the popular pEM4 and pUWL201 shuttle vectors using NGS [7,8, 12,15].

### Highlights

Two closely related *Escherichia coli–Streptomyces* shuttle vectors were sequenced and analysed. Updated sequences and vector maps for pEM4 and pUWL201 facilitate the incorporation of these plasmids into modern synthetic biology workflows.

## Methods

### Bacterial strains, growth conditions and plasmid extraction

*E. coli* JM109 (Promega) was used to propagate plasmids for sequencing analysis. *E. coli* JM109 was grown in Lysogeny Broth (Miller formulation) or on solid lysogeny broth agar plates. Plasmids were introduced into chemically competent *E. coli* JM109 cells by standard procedures [16]. For *E. coli* strains harbouring plasmids, ampicillin was added at a final 100 µg ml^−1^ concentration.

Plasmid DNA was extracted from *E. coli* JM109 cells using the Wizard Plus SV Minipreps DNA Purification System (Promega Catalogue No. A1460) following the manufacturer’s instructions. The plasmid was eluted in nuclease-free water. Following the manufacturer’s instructions, plasmid concentration was measured via a Cytation BioTek Take3 plate reader (Agilent Technologies). Sufficient plasmid purity for whole plasmid sequencing was determined by a 260/230 absorbance ratio of 2.0–2.2 and a 260/230 absorbance ratio of ≥1.8.

### Next-generation plasmid sequencing

The plasmid DNA samples were diluted to a final concentration of 40–65 ng µl^−1^, and aliquots of ≥35 µl were submitted for complete plasmid sequencing. Whole plasmid verification was performed at the Massachusetts General Hospital Center for Computational and Integrative Biology (MGH-CCIB) DNA Core (Cambridge, MA). Sequencing was carried out on an Illumina MiSeq platform with V2 chemistry. Plasmid sequences were assembled using MGH CCIB’s *de novo* assembler UltraCycler v1.0 (Brian Seed and Huajun Wang, unpublished).

## Results and discussion

### Sequencing and analysis of pEM4 and pUWL201

**Fig. 1.**
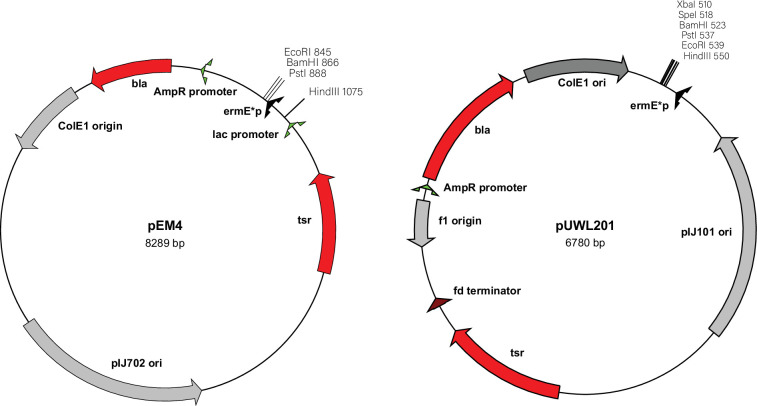
Plasmid maps of pEM4 and pUWL201. The *Streptomyces* and *E. coli* origins of replication are coloured silver. The drug resistance markers for *bla^R^* and *tsr^R^* are coloured red.

Plasmids pEM4 and pUWL201 were sequenced via the Illumina MiSeq NGS platform. pEM4 was sequenced with 209 963 reads, and pUWL201 was sequenced with 12 699 reads. pEM4 is a high-copy number, *E. coli–Streptomyces* spp. shuttle vector, which is derived from pWHM4 *via* insertion of the strong ermE*p promoter from *Saccharopolyspora erythraea* [17]. The ermE*p promoter (i.e. the ‘*ermE-*up’ promoter) is a mutated version of the *ermE* promoter that features a TGG deletion of the *ermE* P1 promoter [18,19]. pEM4 (GenBank Accession No. MN970094) was determined to be an 8289 bp plasmid, which features an additional 1089 bp of sequence as compared to the initially reported plasmid size of 7200 bp [6]. The extra bp of the sequence include the ermE*p promoter and sequence encoding a partial ATP-binding cassette transporter from pIJ486 carried over from the original cloning scheme. pEM4 contains the *bla* gene encoding ampicillin resistance, the *tsr* 23S-rRNA methyltransferase from *Streptomyces azureus* encoding thiostrepton resistance, the high-copy-number ColE1 origin of replication from pUC19 and the pIJ101 origin of replication for *Streptomyces* spp. (Table 1 and Fig. 1).

**Table 1.** Plasmid features of pEM4 and pUWL201

Plasmid features	pEM4	pUWL201
Size (bp)	8289	6780
G+C content (%)	62.6	57.8
ColE1 origin	6901…7583	6383…285
pIJ101 origin	3847…5433	1052…2422
*bla* resistance	7678…249	5626…6285
*tsr* resistance	1607…2416	3564…4373
ermE*p promoter	892…933	633…671
Polylinker	846…1073	509…632
LacZ alpha	688…756	4772…4840

—Feature is not present in the vector.

pUWL201 is another high-copy, *E. coli–Streptomyces* shuttle vector derived from pIJ4070 via introducing a 280 bp fragment encoding the ermE*p promoter into the *Kpn*I–*Xba*I sites of the polylinker region [5]. pUWL201 was determined to be 6780 bp in size, which is 380 bp larger than the originally reported plasmid size of 6400 bp. Sequencing of the MCS revealed the presence of one additional *Xba*I restriction site at position 618 in addition to the site in the polylinker. pUWL201 was determined to have the *bla* gene encoding ampicillin resistance, the *tsr* 23S-rRNA methyltransferase from *S. azureus* encoding thiostrepton resistance, the ColE1 origin of replication for *E. coli* and the pIJ101 origin of replication for *Streptomyces* spp. (Table 1 and Fig. 1). The plasmid also features the fd phage terminator from bacteriophage fd [20]. The fd terminator arrests transcription of the upstream *tsr* gene. The bacteriophage f1 origin of DNA replication is from bacteriophage f1 and initiates and terminates viral ssDNA synthesis [21]. Using a helper phage, the f1 origin facilitates the packaging of ssDNA into phage particles [21]. The f1 origin does not interfere with plasmid replication or play any role without a helper phage.

## Summary

The sequences for the broadly used pEM4 and pUWL201 *E. coli–Streptomyces* shuttle vectors were determined via NGS and analysed. Both vectors revealed additional nucleotide sequences and were larger in size than previously reported. The characterisation of these vectors reveals that vectors traditionally used for the genetic engineering of prokaryotes may contain carryover sequences not explicitly required for plasmid function. This work should facilitate efforts to generate ‘minimal’ versions of these *E. coli–Streptomyces* spp. vectors and will enable their adaptation to synthetic biology.

## Author Notes

The raw sequence reads are included as a supplementary file with the online version of the article.

## Abbreviations

ATP: adenosine triphosphate
LA: lysogeny broth agar
LB: lysogeny broth
MCS: multiple cloning site
NGS: next-generation sequencing
ssDNA: single-stranded deoxyribonucleic acid
WGS: whole genome sequencing
